# Supplementary material for: The use of mixed collagen-Matrigel matrices of increasing complexity recapitulates the biphasic role of cell adhesion in cancer cell migration: ECM sensing, remodeling and forces at the leading edge of cancer invasion
Source: PLoS One. 2020 Jan 16;15(1):e0220019. doi: 10.1371/journal.pone.0220019 (PMC6964905; doi:10.1371/journal.pone.0220019)
Supplement: S1 Table — (DOCX) [file pone.0220019.s001.docx]

**S1 Table.** Main SAENO software parameter used for TFM acquisition.

| NAME | VALUE | DESCRIPTION |
| --- | --- | --- |
| MODE | Regularization | Unconstrained force reconstruction |
| BEAMS | 300 | Number of fibers per tetrahedron |
| K0 | 300 (C, CM), 900 (CM+) | Linear stiffness parameter |
| D0 | 0.0008 | Buckling coefficient of the fibers |
| Ls | 0.02 (C, CM), 1 (CM+) | Onset of fiber strain-stiffening |
| Ds | 0.3 (C, CM), 1 (CM+) | Strain-stiffening coefficient of the fibers |
| DRIFT CORRECTION | 1 | Performs drift correction |
| DRIFT STEP | 2.0 e-06 | Initial step width with which the optimal drift value is searched (m) |
| DRIFT RANGE | 30 e-06 | Maximum search range for drift correction |
| ALLIGNSTACK | 1 | Perform a z-dependent drift correction |
| FIBER PATTERN MATCHING | 1 | Calculate deformations |
| SUBPIXEL | 0.0005 | Convergence criterion in voxels of the fiber matching algorithm. |
| VB MINMATCH | 0.7 | Only sections, which are matched with a higher cross-correlation than this value, are considered valid for force reconstruction |
| VB SX | 12 | Size of cross-correlated sections in x direction (voxels) |
| VB SY | 12 | Size of cross-correlated sections in y direction (voxels) |
| VB SZ | 12 | Size of cross-correlated sections in z direction (voxels) |
| BOXMESH | 1 | Build a mesh |
| BM_GRAIN | 7.5 e-06 | Grid constant of the regular cubic finite element mesh (m) |
| BM_N | 54 | Number of nodes in every dimension (x,y,z). |
| BM_MULOUT | 1.2 | Stretch the outer nodes away from the center  by this factor |
| BM_RIN | 160e-6 | Stretch only nodes that are farther away from  the center than this value (m) |
| ALPHA | 3.0e9 | Regularization parameter in squared Newton per square meter |
| REG_ITERATIONS | 50 | Maximum number of iterations |
| REG_CONV_CRIT | 0.01 | The algorithm stops if the relative standard  deviation of L in the last 6 iterations falls below  this value |
| REG_SOLVER_STEP | 0.33 | Step width parameter of the regularization |
| FM_RMAX | 100e-6 | Only forces that lie within this range (m) from the center are considered for the analysis |
