# Supplementary material for: The use of mixed collagen-Matrigel matrices of increasing complexity recapitulates the biphasic role of cell adhesion in cancer cell migration: ECM sensing, remodeling and forces at the leading edge of cancer invasion
Source: PLoS One. 2020 Jan 16;15(1):e0220019. doi: 10.1371/journal.pone.0220019 (PMC6964905; doi:10.1371/journal.pone.0220019)
Supplement: S2 Table — (DOCX) [file pone.0220019.s002.docx]

**S2 Table**. Concentration of growth factors in our experiments and the bibliography.

| **Growth Factors** |  | **CM** |  | **CM+** |  | **R&D (ED_50_)** |  | **PeproTech (ED_50_)** |
| --- | --- | --- | --- | --- | --- | --- | --- | --- |
| EGF (ng/ml) |  | ~0,01-0,03 |  | ~0,02-0,06 |  | 0,02-0,1 |  | ≤1 |
| bFGF (ng/ml) |  | ~0-3e^-6^ |  | ~0-6e^-6^ |  | 0,1-0,6 |  | ≤5 |
| NGF (ng/ml) |  | ~5e^-3^ |  | ~0,01 |  | 0,2-2 |  | ≤1 |
| PDGF (ng/ml) |  | ~3e^-4^ |  | ~6e^-4^ |  | 1,5-6 |  | ≤1 |
| IGF-1 (ng/ml) |  | ~0,39 |  | ~0,78 |  | 0,3-1,5 |  | 6 |
| TGFB-b (ng/ml) |  | ~0,05 |  | ~0,114 |  | 0,04-0,2 |  | 0,05-0,2 |

Average concentration of main soluble growth factors found in Matrigel within hydrogels C, CM, and CM+ (Data were obtained from the Corning website according to its production lot). Effective dose 50 (ED_50_) determined by cell proliferation assays in human cancer cells (Data were obtained from the R&DSystems and PeproTech websites).
