# Supplementary figures and images for: The use of mixed collagen-Matrigel matrices of increasing complexity recapitulates the biphasic role of cell adhesion in cancer cell migration: ECM sensing, remodeling and forces at the leading edge of cancer invasion
Source: PLoS One. 2020 Jan 16;15(1):e0220019. doi: 10.1371/journal.pone.0220019 (PMC6964905; doi:10.1371/journal.pone.0220019)

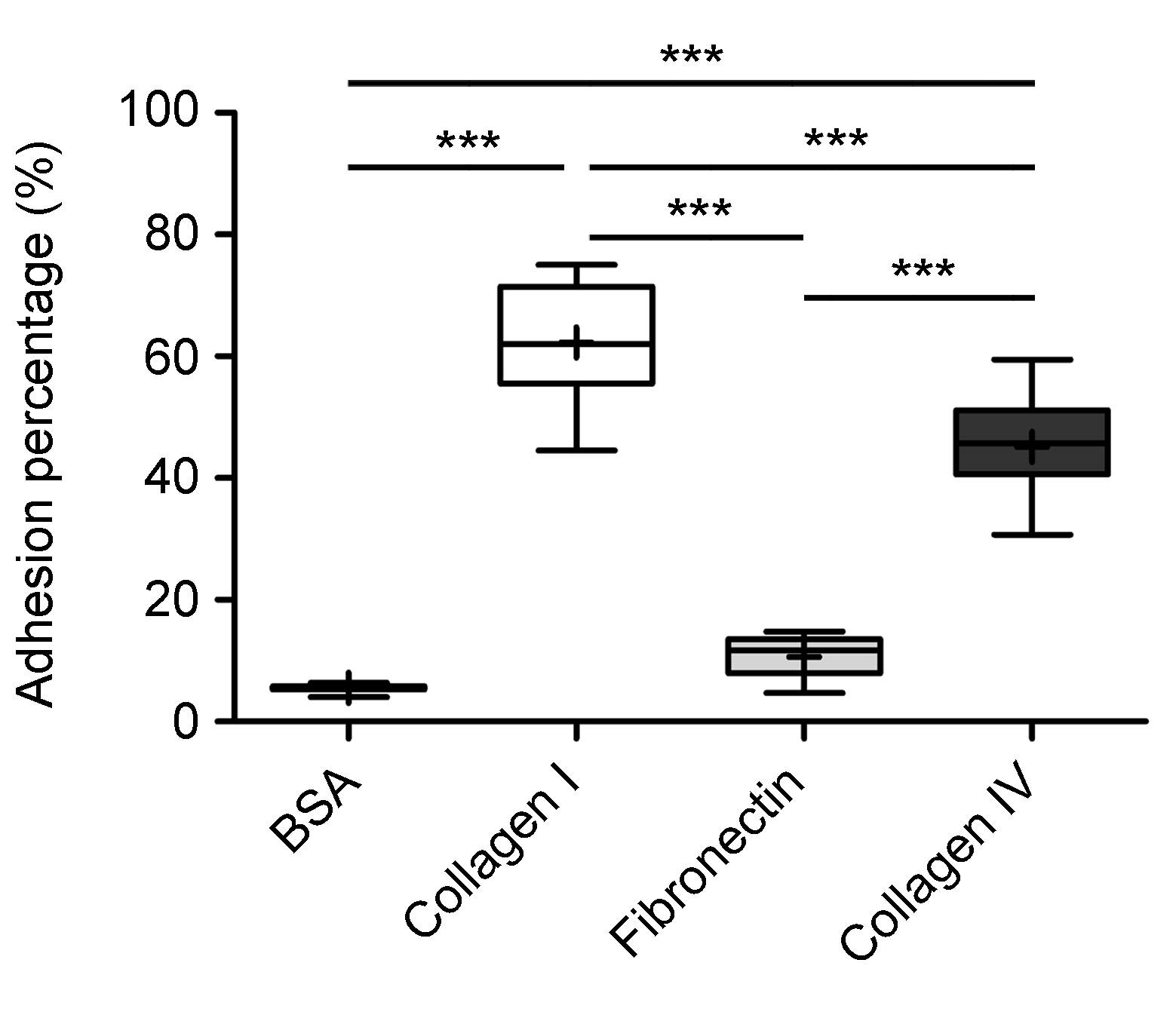

Supplement: S1 Fig — H1299 cells were seeded over collagen I, collagen IV and fibronectin coatings. BSA was used as negative adhesion control. *** Indicates highly statistically significant difference between groups (p<0.001) compared by one-way ANOVA analysis. Four wells were analyzed per ECM protein and experiment. The experiment was performed in triplicate. (TIF) [file pone.0220019.s003.tif]
