# Supplementary material for: The use of mixed collagen-Matrigel matrices of increasing complexity recapitulates the biphasic role of cell adhesion in cancer cell migration: ECM sensing, remodeling and forces at the leading edge of cancer invasion
Source: PLoS One. 2020 Jan 16;15(1):e0220019. doi: 10.1371/journal.pone.0220019 (PMC6964905; doi:10.1371/journal.pone.0220019)
Supplement: Attachment — Submitted filename: review-Anguiano.docx [file pone.0220019.s013.docx]

The manuscript from Anguiano describing H1299 lung cancer cells behaviour in various collagen/matrigel matrices is interesting and valuable despite its mainly technological and descriptive approach. Experiments are generally rigorously performed and analysed. The main point addressed by the authors is the impact of matrix stiffness on cell behaviour, however, the use Matrigel raises important questions because it introduces a bias linked to its bio-active composition (growth factors and ECM proteins). This could have influenced any of the parameters analysed in the study (migration, MMPs, integrin expression, FAs, filopodia..) except those concerning stiffness measurements of the acellularized scaffolds. The model is interesting but the parameters affecting the cells must be considered. Authors mention “small sized focal adhesions and larger focal adhesions” in their abstract but such adhesion structures are not shown, neither quantified. Filopodia do not belong to focal adhesions.

Authors should take these comments in consideration all the way through their manuscript abstract and should adapt their interpretations accordingly.
